# T cell Activation Marker HLA-DR Reflects Tacrolimus-Associated Immunosuppressive Burden and BK Viremia Risk After Kidney Transplantation – An Observational Cohort Study

**DOI:** 10.3389/ti.2025.14443

**Published:** 2025-07-17

**Authors:** Simon Aberger, Max Schuller, Agnes A. Mooslechner, Konstantin A. Klötzer, Barbara Prietl, Verena Pfeifer, Alexander H. Kirsch, Alexander R. Rosenkranz, Katharina Artinger, Kathrin Eller

**Affiliations:** ^1^ Division of Nephrology, Department of Internal Medicine, Medical University of Graz, Graz, Austria; ^2^ Department of Internal Medicine I, Nephrology, Paracelsus Medical University, Salzburg, Austria; ^3^ Otto Loewi Research Center, Division of Pharmacology, Medical University of Graz, Graz, Austria; ^4^ Center for Biomarker Research in Medicine, Graz, Austria; ^5^ Division of Endocrinology and Diabetology, Department of Internal Medicine, Medical University of Graz, Graz, Austria

**Keywords:** immune monitoring, immunosuppression, kidney transplantation, translational nephrology, personalized medicine

## Abstract

Kidney transplantation (KT) is the current treatment of choice in patients with end-stage kidney disease. Immunosuppression is required to prevent acute rejection but is associated with a high incidence of adverse events. The immunosuppressive burden substantially differs between individuals, necessitating new immune monitoring strategies to achieve personalization of immunosuppression. To compare the evolution of T cell profiles in correlation with immunosuppression and clinical outcomes, 87 kidney transplant recipients were followed for 12 months after KT. Flow cytometry along with assessment of T cell activation markers and clinical data was performed before KT and during study visits 10 days, 2 months and 12 months after KT. Longitudinal T cell phenotyping revealed a significant decrease of T cell activation markers HLA-DR, FCRL3, and CD147 in CD4^+^ effector T cells after KT. The most pronounced reduction (75%) was found for the activation-proliferation marker HLA-DR, which persisted throughout the observational period. The decrease in HLA-DR expression reflected immunosuppressive burden through strong associations with tacrolimus trough-level exposure (coeff = −0.39, p < 0.01) and BK viremia incidence (coeff = −0.40, p < 0.01) in multivariable regression analysis. T cell activation marker HLA-DR emerges as a potential biomarker for tacrolimus-related immunosuppressive burden in association with BK viremia risk following KT.

## Graphical Abstract

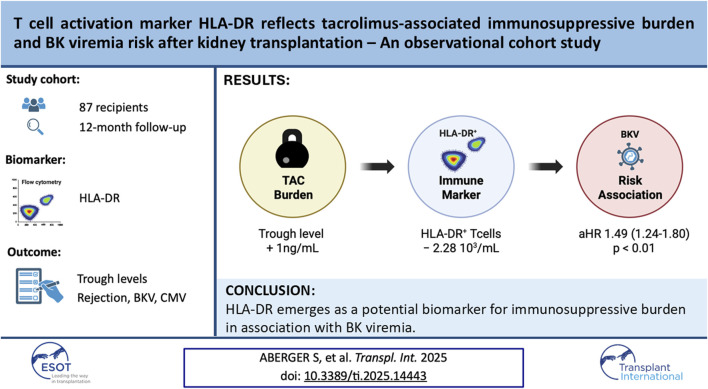

## Introduction

Kidney transplantation (KT) is the current treatment of choice in patients with kidney failure due to survival benefit and improved quality of life. Despite the administration of high-dose immunosuppressive therapy, acute rejection still affects over 10% of kidney transplant recipients (KTR) within the first 12 months [1]. Prolonged or repeated exposure to high-dose immunosuppression is associated with frequent adverse events including metabolic complications, susceptibility to infections and increased risk of malignancy [2]. The trough-level-guided use of calcineurin inhibitors is the cornerstone of T cell suppression in most immunosuppressive regimens. However, the biologically evident level of immunosuppression may vary substantially between individual patients. This variability demands biological effect measures to monitor the overall fitness of the immune system and guide treatment decisions in post-transplant care.

Assessment of the individual immune profile by immune cell phenotyping is currently emerging as a research field with prospects in autoimmunity, oncogenesis, and transplantation [3]. Single-cell sequencing and spatial transcriptomics of kidney allograft biopsies have been used to elucidate cellular interplay in acute rejection after KT, showing CD4^+^ and CD8^+^ T effector cells (T_eff_) as well as innate immune cells (i.e., natural killer cells) expressing a variety of activation markers (i.e., FcγRIII, FCRL3, CD25, HLA-DR) [4, 5]. In peripheral blood CD4^+^ and CD8^+^ T_eff_ these activation markers have been shown to correlate with antigen-induced proliferation (i.e., HLA-DR) [6] and acute rejection (i.e., CD28, HLA-DR) [7, 8]. On the other hand, CD4^+^ and CD8^+^ T cells over-expressing markers of T cell senescence (i.e., TIGIT, LAP) [9, 10] correlate with exhaustion of donor-specific effector T cells positively impacting long-term graft tolerance [11], while activated regulatory CD4^+^ T cells exert tolerogenic effects already early after KT [12]. The biological effect of tacrolimus has been demonstrated to significantly impact the differentiation and proliferative capacity of CD4^+^ T cell populations [13], making CD4^+^ T cells a potential surrogate marker for CNI-associated immunosuppressive burden in translational research. Other immune markers include Torque Tenov viral load starting 2–3 months after KT [14]. However, appropriate markers especially during the first 8 weeks after KT are still missing.

There is currently a lack of comprehensive data regarding differential biological effects of immunosuppressants on T cell profiles following transplantation. Exploring these changes may i) help to individualize CNI prescription in difficult-to-treat patient subgroups and ii) identify T cell markers correlating with immunosuppressive burden, which could be used as new immune monitoring tools after KT. We therefore chose to conduct a prospective, biologic effect study in a cohort of kidney transplant recipients (KTR) by correlating pharmacological data and clinical outcomes with longitudinal phenotyping of T cell activation markers before and after KT.

## Materials and Methods

### Study Design and Population

A longitudinal, single-center cohort study evaluating immune cell subpopulations and short-term post-transplant outcomes in 87 KTR was conducted. The study was designed to prospectively enroll low-immunological risk KTR between September 2017 and August 2020 [15] (Study flowchart: Supplementary Figure S1). Patients receiving immunosuppression within the past 3 months, AB0-incompatible KT, repeated KT and high immunological risk patients were not included in the study (exclusion criteria are further detailed in the Supplementary Material). All patients received basiliximab or ATG, prednisone, mycophenolate, and tacrolimus per standardized protocols. Blood sampling and clinical data collection were performed pre-transplant (preKT), and at 10 days (D10), 2 months (M2), and 12 months (M12) post-transplant. Complete follow-up was obtained for 87 patients to perform a cohort analysis. The study protocol was approved by the Ethics Committee of the Medical University of Graz, Austria (ID 28-514 × 15/16).

### T cell Phenotyping

Flow cytometry was conducted on peripheral blood mononuclear cells (PBMCs) isolated from whole blood samples, collected at study visits. Purified cells were stained with selected monoclonal antibodies (Supplementary Table S1) with BD LSR Fortessa Flow Cytometer (BD Biosciences, USA). T cell phenotyping included CD4^+^ regulatory T cells (T_reg_) defined as CD3^+^CD4^+^CD127^-^Foxp3^+^ according to OMIP-053 by Nowatzky et al [16], considering the interaction of T_reg_ marker CD25 with anti-CD25 antibody basiliximab [17]. CD4^+^ effector T cells (T_eff_) were conventionally defined as CD3^+^CD4^+^CD25^−^CD127^+^CD45RA^−^ and confirmed as being Foxp3^-^ (Supplementary Figure S2). Our selected antibody panels reflecting T cell activation status (including FCRL3, HLA-DR, CD147, CD15s, Ki67) were then separately studied on CD4^+^ T_reg_ and T_eff_ populations (Supplementary Table S2). Gating and exploration of data using tSNE (t-distributed stochastic neighbor embedding) and FlowSOM/ClusterExplore algorithm were done by FlowJo analysis software (BD Biosciences, USA).

### Tacrolimus Data

Tacrolimus dose and trough levels (TL) were recorded weekly to biweekly during the first 12 weeks after KT and at M12. Therapeutic drug monitoring of tacrolimus TL was performed by a validated LC-MS/MS assay. Tacrolimus TL targets were 8–10 ng/mL during the first 2 months and 6–9 ng/mL thereafter. The high granularity of tacrolimus TL data during the first 12 weeks after KT was transposed into a TL trendline. Tacrolimus-associated immunosuppressive burden was then estimated as the area under the curve (AUC) of the tacrolimus TL trendline by trapezoidal rule [18]. This estimate of cumulative tacrolimus TL exposure referred to as “TL AUC” throughout the manuscript.

### Clinical Data

Occurrence and clinical data of biopsy-proven acute rejection (BPAR; using Banff 2019 classification [19]), CMV viremia (defined as ≥100 copies/mL), and BK-viremia (defined as ≥ 200 copies/mL) were documented at each study visit. Screening for viremia was done according to local practice guidelines every 7–14 days during the first two months, followed by readings every other month during the first year after KT. KTR with CMV D^+^/R^−^ status received prophylaxis for 6 months, otherwise a preemptive strategy was followed. Kidney biopsies were performed by indication and at the local physician’s discretion only.

### Statistical Analysis

Baseline characteristics were summarized using descriptive analysis with mean ± standard deviation (SD) or median with interquartile range (IQR) for continuous variables and frequency tables for categorical variables. Continuous variables were tested for normality with Shapiro–Wilk tests and QQ plots. Parametric and non-parametric tests were used for group comparison where appropriate, with multiplicity adjustment by Holm-Sidak method. For the longitudinal assessment of T cell counts, a linear mixed-effects model was fitted using restricted maximum likelihood (REML) estimation, including time as a fixed effect and patients as random intercepts. Spearman correlation coefficient was used to assess the simple relationships between the independent variables TL AUC and T cell counts.

To further explore the underlying immunologic and pharmacologic relationships in a translational approach, we first assessed whether tacrolimus exposure (TL AUC) was associated with immune activation by modeling HLA-DR^+^ T_eff_ counts as a dependent variable in a multivariable linear regression, with TL AUC as the main predictor. A cox regression was then used to assess whether HLA-DR^+^ T_eff_ counts were associated with outcomes (BKV, CMV, BPAR) independent of TL AUC. The proportional hazards assumption using Schoenfeld residuals was confirmed. HLA-DR^+^ T_eff_ counts measured on day 10 and month 2 post-transplant were modeled as time-dependent covariates, corresponding to event occurrence before and after month 2, respectively. Multivariable models were adjusted for immunosuppression-related confounders with known associations with both the exposures (tacrolimus exposure, T cell counts) and outcomes (BKV, CMV, BPAR), including induction agent, CNI formulation, mean mycophenolate mofetil dose, and cumulative steroid exposure. In addition, we assessed univariable associations of donor- and recipient-related characteristics. Among these, age, sex and KDRI met the inclusion threshold (p <0.20) and were retained in multivariable models to balance clinical relevance with statistical parsimony to minimize overfitting. Time-dependent receiver operating characteristic (tdROC) curve was used to determine the predictive capability and cutoff of T cell counts for BK viremia risk. BK viremia incidence was then displayed by Kaplan-Meier curves above and below the predictive cutoff of day 10 (prior to any event) with log-rank test. All statistical analysis and data visualization was done with R Statistical language (version 4.3.2; R Foundation for Statistical Computing, Vienna, Austria). The following packages were utilized: “tidyverse”, “lme4”, “survminer”, “survival”, “Evalue” and “ggplot2”. A p-value <0.05 was considered statistically significant.

## Results

### Characteristics of the Study Cohort

Recipients were of Caucasian ethnicity (>90%), with a male preponderance (63%) and a median pretransplant dialysis vintage of 29 months (Table 1). The median recipient age was 59 years, the mean recipient BMI was 27 and the median KDRI was 1.15 (Table 1). Patients received basiliximab (94.3%) or low-dose ATG (5.7%) for induction, with an initial tacrolimus daily-dose of 0.1 mg/kg, alongside corticosteroids and mycophenolic acid for maintenance by standard protocol. Patients receiving ATG tended to be younger with a higher number of HLA-mismatches (Supplementary Table S3). Mean tacrolimus TL was 10.2 (±3.1) ng/mL at day 10, decreasing to 6.3 (±1.3) ng/mL by M12 (Supplementary Table S4). Recorded events included BPAR n = 16 (15 TCMR, 1 mixed TCMR-ABMR, median time-to-event 14 days), BKV n = 21 (median peak-level 1.1 log^4^ and time-to-event 59 days) and CMV n = 48 (median peak-level 1.3 log^3^ and time-to-event 67 days), (Supplementary Table S5; Supplementary Figure S7).

**TABLE 1:** Donor and recipient characteristics with immunosuppressive regimes are presented as mean (M) ± standard deviation (SD) when normally distributed and otherwise as median (MDN) and interquartile range (IQR) or absolute number (N) with relative percentage (%) for the whole cohort.

Recipient characteristics		N = 87
Female N (%) Male N (%)		32 (36.8%) 55 (63.2%)
Age [years] MDN (IQR)		59 (53–66)
BMI [kg/m^2^] MDN (IQR)		27.9 (23.6–29.1)
Hemodialysis		71 (82%)
Peritoneal dialysis		13 (14.7%)
Preemptive transplantation		3 (3.3%)
Dialysis vintage [mo] (MDN ± IQR)		29 (24–35)
Diabetes mellitus		16 (18%)
Arterial hypertension		84 (97%)
ADPKD		16 (18.4%)
Ethnicity N (%)		
Caucasian Turkish Asian Other		82 (94%) 2 (2.4%) 1 (1.2%) 2 (2.4%)

Donor characteristics		**N = 87**
Age [years] MDN (IQR)		57.5 (49–67)
BMI [kg/m^2^] MDN (IQR)		26.2 (24.1–28.5)
Expanded-criteria donor		51 (58.6%)
Donor after cardiac death		4 (4.6%)
KDRI MDN (IQR)		1.15 (1.02–1.23)
HLA mismatch N (%)		
0 1 2 3 4 5 6		2 (2.3%) 4 (3.4%) 6 (6.7%) 24 (28.6%) 35 (40.6%) 15 (17.2%) 1 (1.2%)

Immunosuppression		**N = 87**
Induction agent		
Basiliximab		82 (94.3%)
Anti-thymocyte globulin		5 (5.7%)
Maintenance regime		
Glucocorticoids		87 (100%)
Mycophenolic acid		87 (100%)
Tacrolimus	Twice daily	55 (63.2%)
	Once daily	32 (36.8%)

### T cell Activation Marker HLA-DR Identifies Effector T cells Susceptible to Tacrolimus

To identify CD4^+^ T cell subpopulations changing after induction therapy, we compared CD4^+^ T_eff_ and CD4^+^ T_reg_ immediately before transplantation (preKT) and 2 months after transplantation (M2) by unsupervised cluster-based analysis stratified by T cell activation status.

**FIGURE 1:**
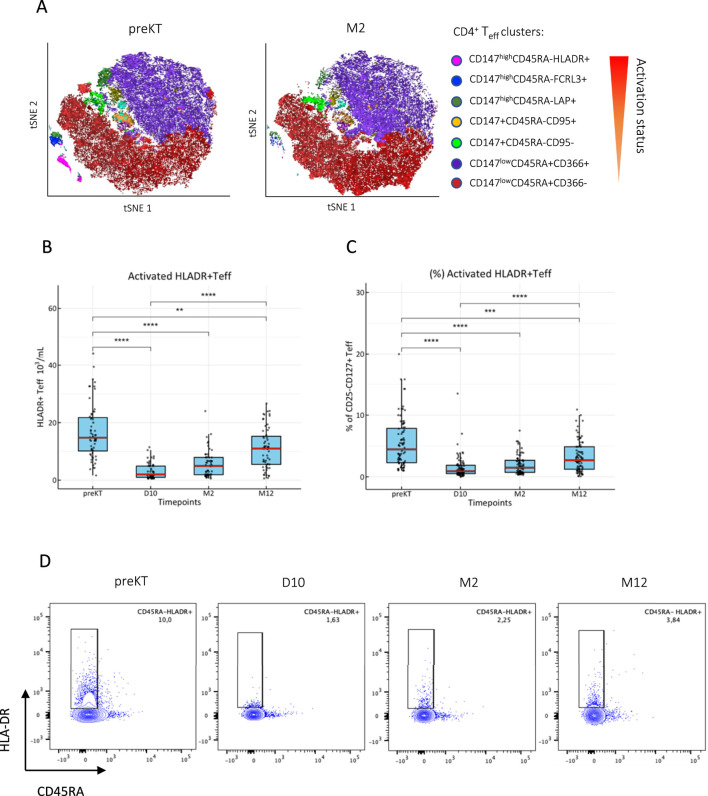
T cell activation marker HLA-DR identifies a CD4^+^ T cell subset susceptible to immunosuppression after KT. **(A)** CD3^+^CD4^+^CD25^−^CD127^+^ T_eff_ were clustered by activation status using FlowSOM algorithm and ClusterExplorer in FlowJo analysis software from peripheral PBMCs isolated immediately before and 2 months after KT. **(B, C)** The longitudinal evolution of absolute cell counts and frequencies are shown as box blots (MDN ± IQR) for all study visits with multiple group comparison by mixed-effects analysis; significant results are shown by asterisks (**) p < 0.01, (***) p < 0.001, (****) p < 0.0001. **(D)** Representative raw flow cytometry contour plots of one patient for each timepoint.

Among CD4^+^ T_eff_, activated clusters expressing activation markers CD147^high^, FCRL3^+^ and HLA-DR^+^ were significantly reduced at M2, while non-proliferating and naive CD45RA^+^ T cell clusters did not change (Figure 1A). Quantitative, longitudinal comparison of T cell subsets identified only HLA-DR^+^ T_eff_ to significantly decrease already at D10 after KT and remain significantly reduced until M12 (Figures 1B–D), while FCRL3^+^ and CD147^high^ T_eff_ returned to baseline by M12 (Supplementary Figure S3). Calculation of the relative change from baseline revealed that the nadir of HLA-DR^+^ T_eff_ counts was reached at D10 (−75.6% from baseline), and cell counts showed an increasing trend at M2 (−64.7% from baseline), however, they remained significantly decreased at M12 (−22.3% from baseline), (Supplementary Table S6).

**FIGURE 2:**
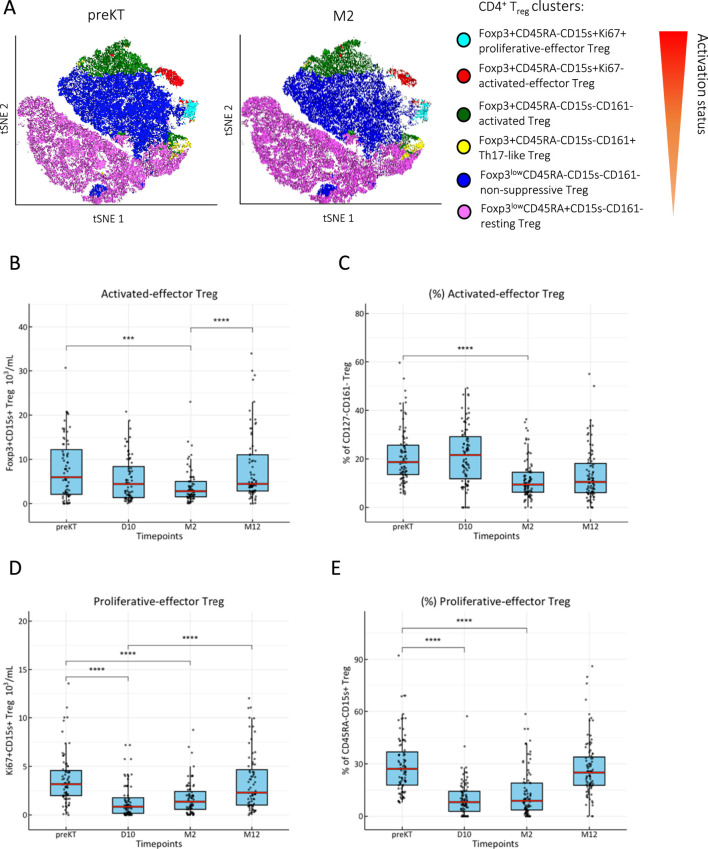
Effector T_reg_ replenish after induction therapy. **(A)** CD3^+^CD4^+^Foxp3^+^CD127^-^ T_reg_ were clustered by activation status using FlowSOM algorithm and ClusterExplorer in FlowJo analysis software from peripheral PBMCs isolated immediately before (preKT) and 2 months after KT (M2). Temporary decrease of absolute counts and frequencies of **(B, C)**: activated CD45RA^−^CD15s^+^ T_reg_ and **(D, E)**: Ki67^+^ proliferative-effector T_reg_ after KT; box blots (MDN ± IQR) for all study visits with multiple group comparison by mixed-effects analysis; significant results are shown by asterisks (***) p < 0.0001, (****) p < 0.00001.

Among CD4^+^ T_reg_, a transient decrease of proliferative and activated Foxp3^+^CD45RA^−^CD15s^+^ effector T_reg_ after KT with a general shift towards a CD45RA^+^CD15s^−^ resting phenotype (Figure 2A) was noted. However, proliferative and effector T_reg_ were fully replenished by M2 or between M2 and M12 (Figures 2B-E), and expression of Foxp3 followed the same trend (Supplementary Figure S4). The known interference of basiliximab with anti-CD25 monoclonal antibodies was evident at D10 and M2 in contrast to patients treated with ATG, however, no major differences were found in Foxp3^+^ T_reg_ and HLADR^+^ T_eff_ subsets (Supplementary Figure S5).

**FIGURE 3:**
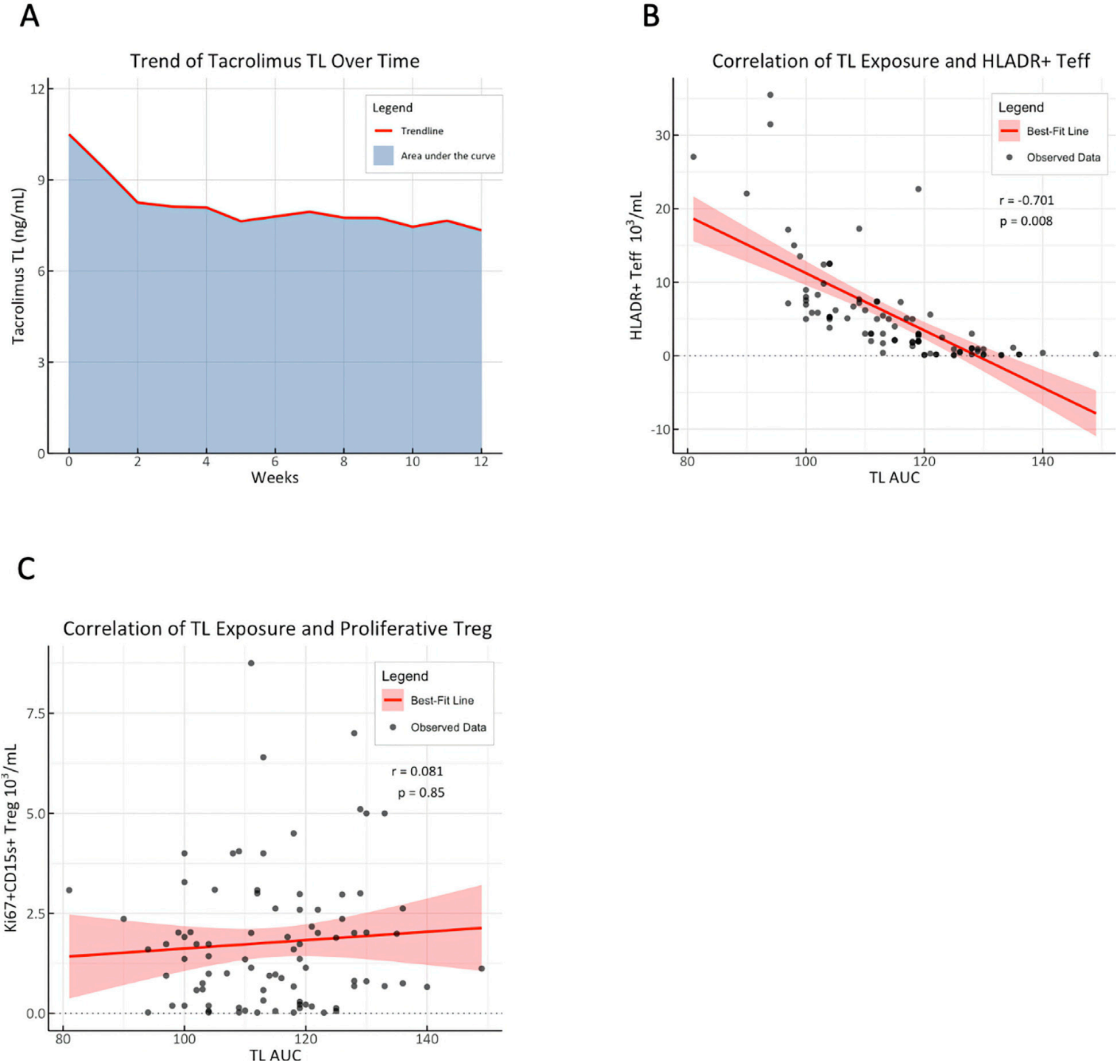
HLA-DR^+^ T_eff_ counts strongly correlate with tacrolimus trough level exposure. **(A)** Median tacrolimus trough level (TL) trend over time is shown as a red line. The area under the curve (AUC) was calculated by the trapezoidal rule (median AUC = 113.7 ng*t/mL) to represent tacrolimus TL exposure. TL exposure (TL AUC) was then plotted against the **(B)**: mean HLA-DR^+^ T_eff_ count and **(C)**: proliferative-effector Treg counts of individual patients starting at D10 until M2; Spearman correlation coefficient (r) was calculated to determine the strength of the relation.

We next sought to explore the sustained decrease in HLA-DR^+^ T_eff_ counts by testing the relation between cell quantity and immunosuppressive burden. Slope analysis of mean tacrolimus TL and HLA-DR^+^ T_eff_ counts over 12 months revealed a decrease of 2.28 × 10^3^/mL cells per 1 ng/mL increase in tacrolimus TL (Supplementary Table S4). A strong negative correlation between tacrolimus burden, estimated as TL AUC (Figure 3A), and the HLA-DR^+^ T_eff_ counts during the first weeks until M2 after KT was observed (r = −0.70, p = 0.008; Figure 3B). To account for potential confounders related to recipient characteristics, donor quality, and the immunosuppressive regimen, we performed multivariable linear regression. The significant association between HLA-DR^+^ T_eff_ counts, and TL AUC remained robust across all models (β-coefficient = −0.39, p = 0.0002), (Table 2; Supplementary Table S7). No correlation was found for proliferative-effector T_reg_ counts (Figure 3C).

**TABLE 2:** HLA-DR^+^ T_eff_ counts adjusted for recipient-, donor- and treatment-related covariates is associated with TL exposure.

Association of HLA-DR + Teff counts and TL exposure				
Model	Coefficient	95% CI	p-value	R-squared
Crude.	−0.419	−0.531 to −0.310	2.613 e-07	0.504
Model 1	−0.433	−0.523 to −0.303	5.021 e-06	0.552
Model 2	−0.403	−0.503 to −0.301	5.020 e-06	0.510
Model 3	−0.390	−0.528 to −0.310	2.612 e-04	0.484

Multivariable linear regression was used to adjust the crude association of HLA-DR^+^ T_eff_ counts (dependent variable) and TL AUC for covariates; Model 1 = adjusted for sex + age; Model 2 = adjusted for Model 1+ KDRI; Model 3 = adjusted for Model 2 + ATG + TAC formulation +mean MMF dose + cumulative prednisolone dose.

### T cell Activation Marker HLADR Is Independently Associated With BK Viremia Risk

**FIGURE 4:**
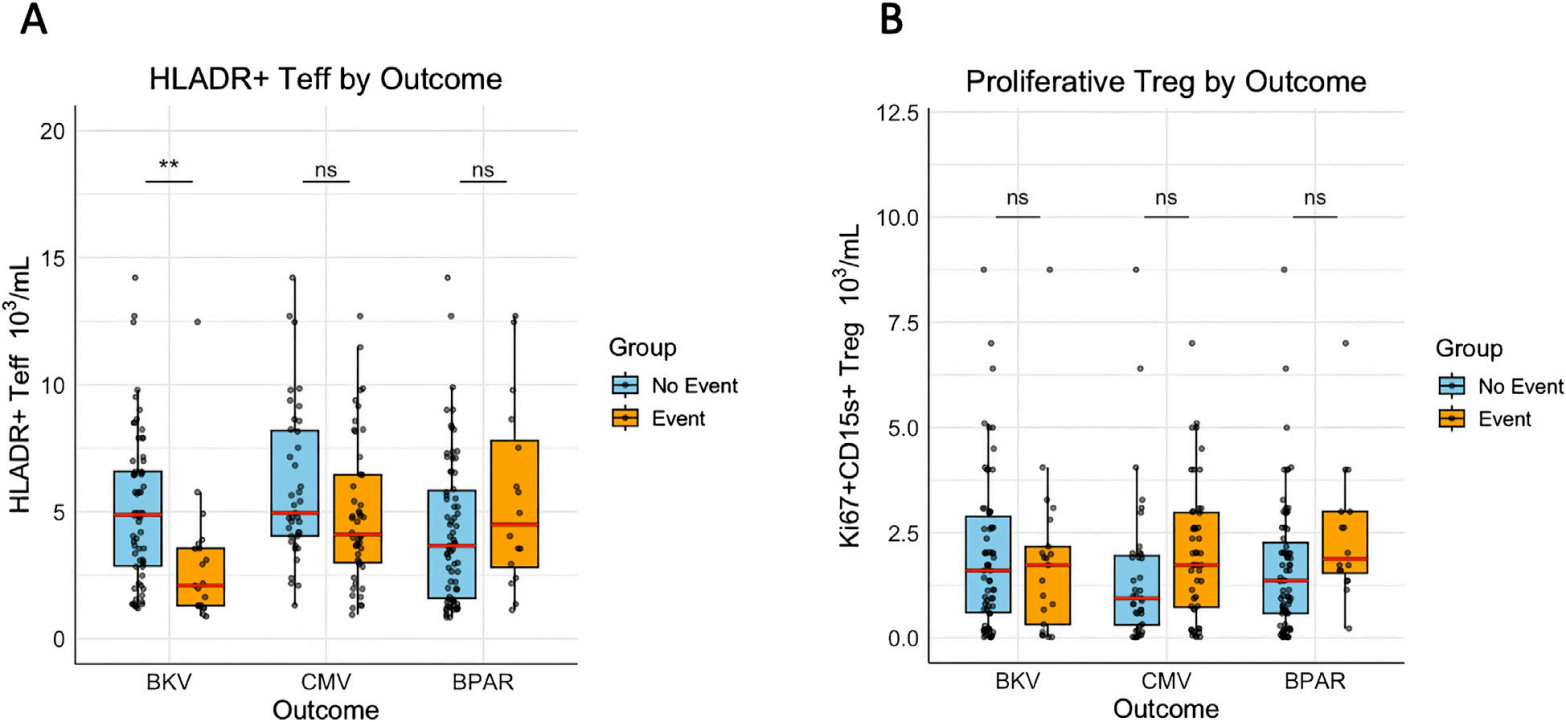
HLA-DR^+^ T_eff_ counts are significantly lower in patients developing BKV viremia. **(A)** The mean HLA-DR^+^ T_eff_ counts and **(B)**: the mean proliferative-effector Treg counts between D10 and M2 of individual patients were pairwise compared between event and no event groups for BKV, CMV, and BPAR. (**) indicates p < 0.01.

We further investigated outcome-oriented associations between the T-cell activation marker HLA-DR and immune-related events, including BK viremia, CMV infection, and BPAR. The HLA-DR^+^ T_eff_ counts were significantly lower in patients who developed BK viremia compared to those who did not (Figure 4A). A similar trend was observed for CMV, although statistical significance was not reached (p = 0.09), while no difference was noted for BPAR (Figure 4A). Again, no difference was found for proliferative-effector T_reg_ counts (Figure 4B). To assess the association between HLA-DR^+^ T_eff_ counts and BKV, CMV, and acute rejection (AR), a time-dependent multivariable cox regression was performed and adjusted for TL AUC and confounders. The significant association between HLA-DR^+^ T_eff_ counts and BKV remained independent from TL AUC and confounders (fully adjusted HR = 1.49, p = 0.00002), (Table 3). No significant associations were identified between HLA-DR^+^ T_eff_ counts and the occurrence of CMV or BPAR (Table 3).

**TABLE 3:** BKV adjusted for TL exposure and covariates is independently associated with HLA-DR^+^ T_eff_ counts.

Association of Outcome variables with HLA-DR + Teff counts					
Outcome	Model	Events/Total (Censored)	Coefficient	HR (95% CI)	p-value
BKV	Crude.	21/87 (66)	−0.717	0.488 (0.31–0.63)	0.00002
	Model 1		−0.425	0.654 (0.51–0.80)	0.0001
	Model 2		−0.377	0.686 (0.57–0.83)	0.0005
	Model 3		−0.402	0.669 (0.55–0.81)	0.0001
CMV	Crude.	48/87 (39)	−0.119	0.88 (0.67–1.17)	0.230
	Model 1		−0.080	0.91 (0.83–1.11)	0.594
	Model 2		0.016	1.03 (0.97–1.09)	0.774
	Model 3		−0.055	0.96 (0.90–1.03)	0.640
BPAR	Crude.	16/87 (71)	0.060	1.04 (0.83–1.20)	0.189
	Model 1		0.058	1.03 (0.86–1.20)	0.189
	Model 2		0.063	1.07 (0.88–1.19)	0.174
	Model 3		0.067	1.08 (0.89–1.20)	0.177

Time-dependent multivariable cox regression was used to test the association of HLA-DR^+^ T_eff_ counts for the outcomes BKV, CMV, and BPAR. The crude model includes only HLADR^+^ T_eff_, Model 1 = adjusted for TL AUC, Model 2 = adjusted for Model 1 + age + sex + KDRI; Model 3 = adjusted for Model 2 + ATG + TAC formulation +mean MMF dose + cumulative prednisolone dose.

**FIGURE 5:**
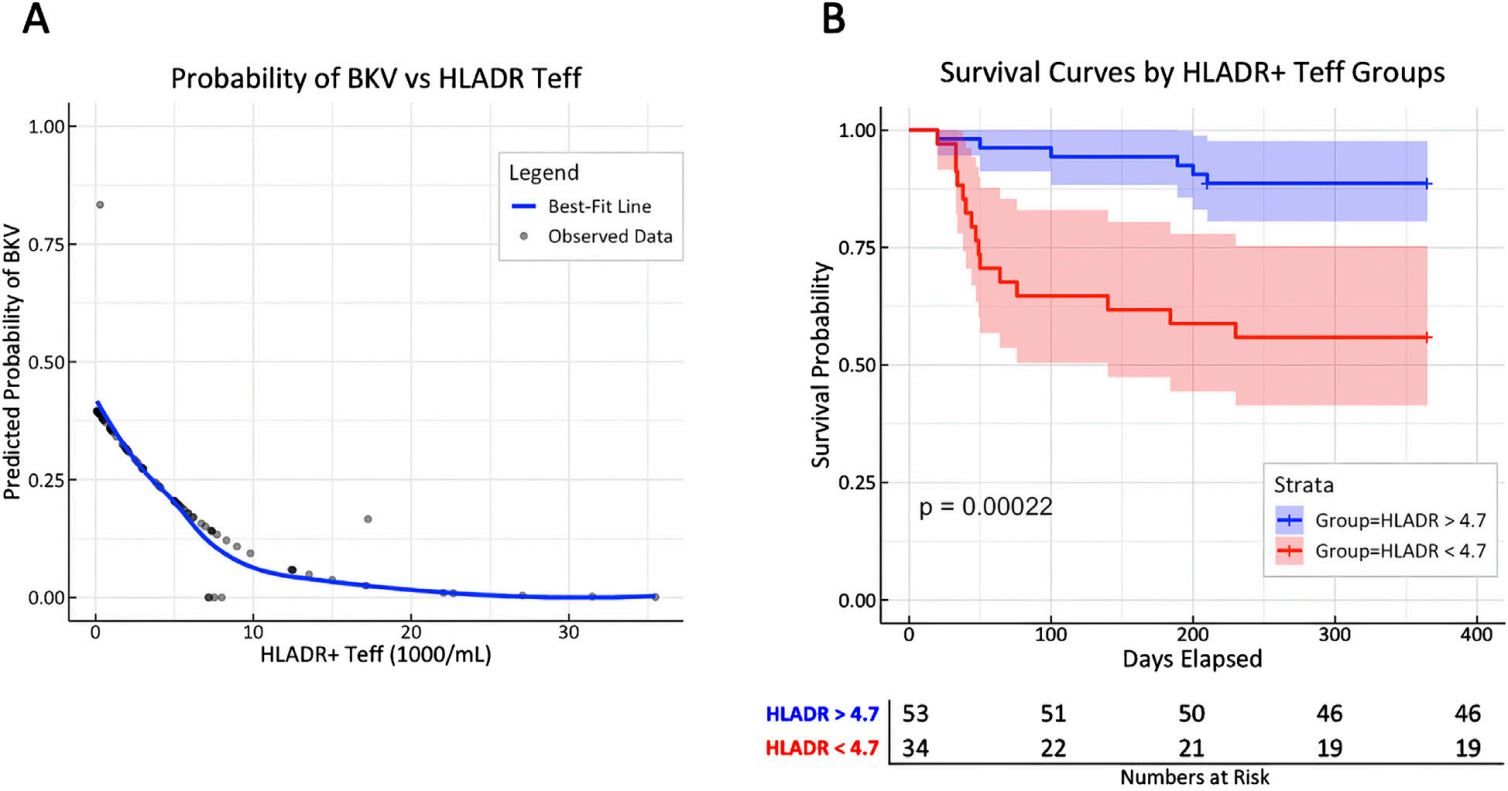
T cell activation marker HLA-DR is associated with BK viremia, potentially allowing risk stratification early after KT. **(A)** Predicted probabilities for the first incident of BK viremia from cox regression stratified by HLA-DR^+^ T_eff_ counts are depicted with a best-fit line (blue line); aHR = 1.49 [1.24–1.80] per unit decrease of HLA-DR, p = 0.00002. **(B)** Patients were stratified by HLA-DR^+^ T_eff_ count at day 10 above or below 4.71 × 10^3^/mL (cutoff determined by tdROC analysis of viremia incidence with AUC of 0.75; p = 0.002) to display the risk difference for experiencing BK viremia by Kaplan-Meier curves with log-rank analysis.

Predicted probabilities of BKV by HLA-DR^+^ T_eff_ counts were modeled from cox regression and depicted with a best-fit line to show the increase in BKV risk with decreasing HLA-DR^+^ T_eff_ counts (Figure 5A). Time-dependent Receiver operating characteristic (ROC) analysis revealed an AUC of 0.75 (p = 0.001), with a specificity of 63% and sensitivity of 85% for an HLA-DR^+^ T_eff_ count of 4.71 × 10^3^ cells/mL at day 10 (Supplementary Figure S6). Stratification of the cohort based on HLA-DR^+^ T_eff_ count above and below this cutoff demonstrated a significant difference in viremia-free survival, as shown by Kaplan-Meier curve analysis (Figure 5B).

## Discussion

This observational cohort study employs longitudinal T cell phenotyping to identify immune markers correlating with immunosuppressive burden and clinical outcomes after KT. The study cohort included 87 prospectively enrolled, immunologically low-risk KTR receiving basiliximab- (94%) or ATG-based (6%) induction therapy with triple immunosuppressive maintenance therapy (steroids, tacrolimus, and mycophenolic acid). Suppression of T cell activation marker HLA-DR was associated with tacrolimus burden and was markedly aggravated in patients developing BK viremia, emerging as a potential immune monitoring tool.

Unsupervised cluster-based analysis of CD4^+^ T_eff_ revealed significant changes among T cell activation markers following KT. The immediate decrease of HLA-DR^+^ CD4^+^ T_eff_ already at D10 indicated an early suppression of T cell proliferation, as HLA-DR expression has been shown to reflect T cell proliferative capacity with antigen stimulation after KT [6]. In contrast, other activation markers, such as FCRL3, and CD147 demonstrated a delayed timeline for observable change. In line with this observation, CNIs have been shown to decrease T cell proliferative capacity in stimulation assays [20]. However, stimulation assays are hampered by frequent preanalytical errors in clinical practice, underscoring the added practical value of using flow cytometry to provide a feasible indicator of the efficacy of immunosuppressive therapy in a real-world setting. In our study, the consistent, inverse relationship between TL exposure and HLA-DR^+^ T_eff_ counts suggests a dose-dependent reduction of T cell quantity. The association between HLA-DR^+^ T_eff_ counts and tacrolimus TL AUC remained robust even after adjusting for potential confounders. Together, these findings support that HLA-DR^+^ T_eff_ count may serve as a surrogate biological measure of a CNI dose-immune effect. Notably, the observable changes in HLA-DR^+^ T_eff_ cell counts within the first two months could complement other immune monitoring tools, such as TTV, which typically exhibit delayed responses to immunosuppression early after KT [21].

Building on this background, the use of tacrolimus TL AUC in our study may provide a more accurate estimation of immunosuppressive burden compared to single or averaged TL measurements. Recent evidence suggests that TL AUC reflects the immunosuppressive burden of CNI-based regimens, with demonstrated correlations to TTV levels and BK viremia risk in a retrospective cohort analysis of kidney transplant recipients [18]. The strong, inverse association between HLADR^+^ T_eff_ cell counts and tacrolimus TL AUC in our study reflects these findings. However, the practical application of TL AUC is limited by its retrospective nature and the need for high data granularity, highlighting the importance of identifying a feasible and reliable surrogate marker for clinical monitoring and adverse event prediction. TTV viral load is currently evaluated as a promising immune monitoring tool after a calibration period of 8 weeks after KT [14].

In our study, the association between BK viremia and HLA-DR^+^ T_eff_ counts remained robust after adjustment for TL AUC and confounding variables. This independent association as early as day 10 after KT is particularly intriguing, given that reduction of immunosuppression remains the mainstay of BKV management and could suggest that early reduction of immunosuppression could mitigate viremia in at-risk patients. Currently, our findings build a biologically plausible association between tacrolimus-based immunosuppression and activated T cell quantity reflected by immune marker HLA-DR, and BK viremia. Based on this association, the observed decrease of 2.28 × 10^3^/mL in HLA-DR^+^ T_eff_ cells per 1 ng/mL increase in tacrolimus TL over time provides valuable pilot data for estimating effect sizes in future studies. However, these findings are preliminary evidence and support the development of prospective investigations to validate and test HLA-DR^+^ T_eff_ count as a biomarker for immunosuppressive burden to mitigate adverse events early after KT.

Previous studies have demonstrated that induction with the anti-CD25 monoclonal antibody basiliximab influences T_reg_ activation markers in CD4^+^ T_reg_ [22], yet without impacting functionality [23]. This was confirmed by the absence of CD25^+^ T_reg_ at day 10 in basiliximab-treated patients, whereas CD25^+^ T_reg_ in ATG-treated patients and Foxp3^+^ T_reg_ in the whole cohort were detectable. Concerning the evolution of Foxp3^+^ T_reg_, we observed a transient decrease of activated and proliferative T_reg_ markers following induction therapy, with reconstitution by month 2 or between month 2 and month 12. Previous studies suggested prognostic relevance of T_eff_/T_reg_ ratio predicting acute rejection after KT [22], however, the reduction of T_eff_ cells was overall stronger than the reduction of T_reg_ in our study. In addition, there was no significant correlation between proliferative T_reg_ subsets and TL-AUC, and no differences were found for clinical outcomes.

From a pathomechanistic view, the stronger association between HLA-DR^+^CD4^+^ T_eff_ cells and BK viremia, compared to CMV infection, is noteworthy. It may reflect fundamental differences in host immune responses, suggesting a critical role of CD4^+^ T cell immunity in the development of BK viremia. This is consistent with emerging strategies to restore BKV-specific immunity, including the use of allogeneic CD4^+^ T-cell therapy [24]. Furthermore, the decrease in HLADR^+^ T_eff_ counts with higher tacrolimus burden and BK viremia risk in our cohort aligns with findings from a previous observational study, suggesting a “CNI-first” approach to immunosuppression reduction as an effective treatment strategy for BK viremia and nephropathy [25]. Contrarily, a more pronounced involvement of CD8^+^ T-cell-mediated immunity in CMV control has been suggested [26], as current investigations into interferon-gamma release assays as a monitoring tool for CD8^+^ cellular immunity aim to guide decisions regarding pre-emptive or prophylactic therapy for CMV [27]. Similarly, TTV viral load is under evaluation as a potential immune monitoring tool for CNI-based immunosuppression, with predictive value for immune-related adverse events [14].

Finally, we identified a predictive threshold for HLA-DR^+^ T_eff_ counts to stratify kidney transplant recipients (KTR) at risk of developing BK viremia. Specifically, an HLA-DR^+^ T_eff_ count below 4.7 × 10^3^/mL at day 10 post-transplantation was associated with meaningful risk prediction for BK viremia (median time to event: 59 days), potentially justifying early adjustment of immunosuppressive therapy. A comparable strategy has been reported in a prospective study, where the pretransplant abundance of CD28^+^ T cells was shown to predict acute rejection risk in patients receiving belatacept (an anti-CD28 monoclonal antibody) compared to tacrolimus [8]. In this regard, our findings remain exploratory and provide preliminary data to support future studies investigating the utility of immune marker-guided CNI dosing and T-cell phenotyping as predictive tools for mitigating viral and immunological complications following kidney transplantation.

Limitations of our study include a small sample size, albeit comparable to other studies in the field. Nonetheless, a total of 348 blood samples for flow cytometry and more than 900 tacrolimus TL data were sufficient for comprehensive analysis. The prospective setting and the use of adjusted regression models to show a dose-immune effect strengthen the internal validity of our study. This analytical strategy was designed to reflect a biologically plausible and mechanistic pathway; however, causality can not be claimed, and residual confounding can not be entirely excluded. For sensitivity analysis, E-value analysis for the adjusted HR of 1.49 for BK viremia was 2.3 (1.8 lower bound), indicating that any unmeasured confounder would need to have a relative risk of at least 2.3 with both HLA-DR expression and BK viremia to fully account for the observed effect. Furthermore, the single-centre design with representation of a central European cohort may limit the overall comparability of our results. Therefore, we acknowledge that our results need further external validation, ideally with additional external cohorts and confirmation by a larger, multicentric trial. We also have to acknowledge that the implementation of flow cytometry may be hampered by technical reproducibility in clinical routine, and a higher frequency of flow cytometric measurements could have improved the granularity of the data. Our study does not include protocol biopsies, *de novo* DSA, tacrolimus single-dose AUC, T cell phenotyping of the CD8^+^ lineage, or T cell stimulation assays, which could be the subject of a follow-up study.

In conclusion, T cell activation marker HLA-DR emerges as a potential biomarker for tacrolimus-associated immunosuppressive burden, yielding a strong association with BK viremia risk following kidney transplantation.

## Data Availability

The original contributions presented in the study are included in the article/Supplementary Material, further inquiries can be directed to the corresponding author.
