# Supplementary material for: T cell Activation Marker HLA-DR Reflects Tacrolimus-Associated Immunosuppressive Burden and BK Viremia Risk After Kidney Transplantation – An Observational Cohort Study
Source: Transpl Int. 2025 Jul 17;38:14443. doi: 10.3389/ti.2025.14443 (PMC12310561; doi:10.3389/ti.2025.14443)

Supplemental Material:

T cell activation marker HLA-DR reflects tacrolimus-associated immunosuppressive burden and BK viremia risk after kidney transplantation – An observational cohort study

Simon Aberger, Max Schuller, Agnes A Mooslechner, Konstantin A Klötzer, Aferdita Gashi, Barbara Prietl, Verena Pfeifer, Alexander H Kirsch, Alexander R Rosenkranz, Katharina Artinger, Kathrin Eller

Capsule Sentence Summary:

In a longitudinal cohort study, T cell activation marker HLA-DR reflected tacrolimus-associated immunosuppressive burden in association with BK viremia risk after kidney transplantation.

STROBE Statement

This manuscript was prepared in accordance with the STROBE (Strengthening the Reporting of Observational Studies in Epidemiology) guidelines for reporting observational studies.

List of exchange plugins for FlowJo™ Version 10.10.0:

- FlowSOM v4.1.0 April 2024, R/Biocoinductor library published Van Gassen et al., Cytometry A, 2015
- ClusterExplorer v1.7.6 May 2023 for data visualization

Unbiased exploration of FACS data was done from CD3+CD4+CD127-Foxp3+CD161- T_{reg} and CD3+CD4+CD25-CD127+Foxp3- T_{eff} mothergates using concatenate function to ensure equal cell sampling and clean representation of data. T-SNE analysis was first done using default settings for knn, perplexity, iterations which were then adjusted according to sample sizes and visual impression from multiple runs. FlowSOM algorithm was used to create metaclusters by assessing multiple runs to reach soft overclustering with default settings for Hierachical Consensus clustering. ClusterExplorer plugin was then used to create a summary plot based on FlowSOM metaclusters including a cluster-based heatmap for differential expression of immune markers.
Isotype controls and the fluorochrome minus one method were used for the quantification of positive signals. Flow cytometric analysis with BD LSR Fortessa was standardized and controlled by system performance checks (BD Cytometer Setup and Tracking Beads) with standardized assay-specific settings using BDOneFlow setup beads (BD Biosciences, USA).

Detailed exclusion criteria:

This longitudinal, observational study aimed to prospectively include low immunological risk patients free of immunosuppressants before receiving a first kidney transplantation. Low immunological risk was defined by the absence of donor-specific antibodies, cPRA < 20% and AB0-compatibility in recipients of a first KT.

- Immunosuppressive medication during the last 3 months before KT
- Immunological high-risk profile
- Maintenance with Belatacept or Cyclosporine
- Repeated KT
- ABO-incompatible donation
- Age < 18 years
- Unable to provide signed, informed consent
- Pregnancy

Supplemental table legend:

Supplemental Table 1. List of Flow cytometry markers to define T cell subsets.
T cell markers were used to subset T cells according to activational status.

Marker	Reported marker	As % of	Phenotype
#1	Foxp3+Treg	Total CD3+CD4 +	CD3+CD4+CD127-FoxP3+
#1a	CD25+Foxp3+ Treg	Total CD3+CD4+	CD3+CD4+CD127-CD25+Foxp3+
#1b	CD127-CD161- Treg	Total CD3+CD4+Foxp3+	CD3+CD4+ CD127-Foxp3+ CD161-
#2	Th17 type Treg	Total CD127-CD161-	CD3+CD4+ CD127-Foxp3+ CD161+
#3	Resting Treg	Total CD127-CD161-	CD3+CD4+Foxp3+CD127-CD161-CD45RA+CD15s-
#4	non suppressive Tc	Total CD127-CD161-	CD3+CD4+Foxp3+CD127-CD161-CD45RA-CD15s-
#5	Activated Treg	Total CD127-CD161-	CD3+CD4+Foxp3^{high}CD127-CD161-CD45RA-CD15s-
#6	Effector Treg	Total CD127-CD161-Foxp3-	CD3+CD4+ Foxp3+CD127-CD161-CD45RA-15s+
#7	Proliferative effector Treg	Total CD45RA-CD15s+	CD3+CD4+ Foxp3+CD127-CD161-CD45RA-CD15s+Ki67+
#9	T effector cells (Teff)	Total CD3+CD4+	CD3+CD4+CD25-CD127+(Foxp3-)
#10	CD366 Teff	Total CD25-CD127+	CD3+CD4+CD25-CD127+CD45RA- CD366+
#11	FCRL3 (CD307c) Teff	Total CD25-CD127+	CD3+CD4+CD25-CD127+ CD45RA-CD307C+
#12	CD147 Teff	Total CD25-CD127+	CD3+CD4+CD25-CD127+ CD45RA-CD147+
#13	PI-16 Teff	Total CD25-CD127+	CD3+CD4+CD25-CD127+ CD45RA-PI-16+
#14	LAP Teff	Total CD25-CD127+	CD3+CD4+CD25-CD127+ CD45RA-LAP+
#15	HLA-DR Teff	Total CD25-CD127+	CD3+CD4+CD25-CD127+ CD45RA-HLA-DR+
#16	CD95 Teff	Total CD25-CD127+	CD3+CD4+CD25-CD127+ CD45RA-CD95+

Supplemental Table 2. List of Flow cytometry antibodies.
List of antibodies used in two distinct panels to define subpopulations and company data. Panel 1 was used to study T_{reg} and Panel 2 for T_{eff}.

Antibody	Catalogue Nr.	Detail	Manufacturer	Panel
FVS	564997	APC-R700	BD Biosciences	1, 2
CD3	560176	APC-H7	BD Biosciences	1, 2
CD4	560650	PerCP-Cy5	BD Biosciences	1, 2
CD25	335824	PE-Cy7	BD Biosciences	1, 2
Foxp3	320208	PE	BD Biosciences	1, 2
CD127	563086	BV510	BD Biosciences	1, 2
CD45RA	563870	BV786	BD Biosciences	1, 2
CD15s	563912	BV421	BD Biosciences	1
CD161	550968	APC	BD Biosciences	1
Ki67	558616	AF488	BD Biosciences	1
CD147	562554	PerCP-Cy5	BD Biosciences	2
CD307c	565026	BB515	BD Biosciences	2
CD95	558814	APC	BD Biosciences	2
HLA-DR	565127	APC-R700	BD Biosciences	2
CD366	565562	BV421	BD Biosciences	2
PI-16	563520	PE	BD Biosciences	2
LAP	562490	PE-CF594	BD Biosciences	2

Supplemental Table 3. Recipient and donor characteristics by induction agent.

Recipient characteristics	Basiliximab (N = 82)	ATG (N = 5)
**Female N (%)**<br>**Male N (%)**	31 (37.8%)<br>51 (62.2%)	1 (20%)<br>4 (80%)
**Age [years] MDN (IQR)**	59 (53-66)	41 (30-55)
**BMI [kg/m2] MDN (IQR)**	27.9 (23.6-29.1)	26.8 (22.4-28.7)
**Diabetes mellitus**	15 (18%)	1 (20%)
**Arterial hypertension**	80 (97%)	5 (100)
**ADPKD**	16 (18.4%)	0
**cPRA < 20%**	82 (100%)	5 (100%)
**Donor characteristics**	**Basiliximab (N = 82)**	**ATG (N = 5)**
**Age [years] MDN (IQR)**	57.5 (49-67)	50 (33-61)
**BMI [kg/m^2] MDN (IQR)**	26.2 (24.1-28.5)	25.9 (19.1-28.9)
**Expanded-criteria donor**	51 (58.6%)	1 (20%)
**Donor after cardiac death**	4 (4.6%)	0
**KDRI MDN (IQR)**	1.15 (1.02-1.23)	1.08 (1.0-1.13)
HLA mismatch N (%)		
0<br>1<br>2<br>3<br>4<br>5<br>6	2 (2.3%)<br>4 (3.4%)<br>6 (6.7%)<br>24 (28.6%)<br>35 (40.6%)<br>11 (17.2%)<br>0	0<br>0<br>0<br>0<br>0<br>4 (80%)<br>1 (20%

Supplemental Table 4. Tacrolimus dose, trough level (TL) and HLA-DR$^+$ T_{eff} counts over time with slope comparison.

Mean Tacrolimus daily dose, tacrolimus TL, and HLA-DR$^+$ T_{eff} counts at study visits. The average slope was calculated for tacrolimus TL data and cell counts to calculate the change in cell count per unit change in tacrolimus TL (2.28 10^3/mL change in cell count per 1 ng/mL change in TL).

Data	Timepoint	N = 87
**Tacrolimus dose [mg/d] M ± SD**	Day 10	6.9 (± 3.4)
	Month 2	5.1 (± 3.7)
	Month 12	3.5 (± 2.1)
**Tacroliimus TL [ng/mL] M ± SD**	Day 10	10.2 (± 3.1)
	Month 2	8.5 ± (1.9)
	Month 12	6.3 ± (1.3)
	Average slope: - 0.34 ng/mL per month	
**HLA-DR$^+$ T_{eff} count [10^3/mL] M ± SD**	Day 10	4.07 (± 2.6)
	Month 2	5.88 (± 4.5)
	Month 12	12.97 (± 7.1)
	Average slope: + 0.76 10^3/mL per month	

Supplemental Table 5. Incidence of outcome measures.
Incidence of viremia incidence with copy numbers and acute rejection with BANFF severity grading on biopsy after KT.

Outcome variable	N = 87
**Polyoma virus (BKV)**	21 (24.1%)
**BKV copies MDN (Min-Max)**	11000 (1230 - 27000)
**Cytomegalo virus (CMV)**	48 (55.2%)
**CMV copies/mL MDN (Min-Max)**	1350 (880 - 3800)
**CMV D+/R- status**	39 (44.8%)
**BPAR**	16 (18.4%)
**BANFF classification**	
Borderline	2 (12.5%)
IA	6 (37.5%)
IB	4 (25%)
IIA	3 (18.75%)
IIB	1 (6.25%)
III	0
**TCMR**	15 (93.75%)
**Mixed TCMR-ABMR**	1 (6.25)

Supplemental Table 6. Absolute and Relative changes of HLA-DR$^+$ T_{eff}, Activated and Proliferative T_{reg} from baseline. The data is derived from the linear mixed-effects model, the p-value is given for the absolute change in cell counts.

Group	Time	Mean count (10^3/mL)	95% CI	Abs. change (10^3/mL)	Rel. change (%)	p-value
**HLADR$^+$ T_{eff}**	preKT	16.68	14.91 - 18.45	REF	REF	-
	d10	4.07	-0.12 - 8.26	-12.62	-75.6	<0.001
	m2	5.88	1.69 - 10.07	-10.8	-64.7	<0.001
	m12	12.97	8.78 - 17.15	-3.72	-22.3	0.003
**Activated T_{reg}**	preKT	7.84	6.44 - 9.24	REF	REF	-
	d10	6.24	2.85 - 9.63	-1.6	-20.4	0.114
	m2	3.82	0.43 - 7.2	-4.02	-51.3	<0.001
	m12	8.04	4.65 - 11.42	0.2	2.5	0.845
**Proliferative T_{reg}**	preKT	3.74	3.19 - 4.28	REF	REF	-
	d10	1.44	0.15 - 2.72	-2.3	-61.5	<0.001
	m2	1.73	0.45 - 3.02	-2.0	-53.6	<0.001
	m12	3.32	2.03 - 4.61	-0.42	-11.2	0.272

Supplemental Table 7. Univariate associations of recipient- and donor-characteristics for variable selection. Regression analysis was performed to investigate the univariable associations of potential covariates.

ASSOCIATIONS BETWEEN HLA-DR+ TEFF AND VARIABLES			
VARIABLE	Coefficient	p-value	R-squared
AGE	0.111	0.094	0.212
SEX	-0.582	0.189	0.030
KDRI	-1,297	0.102	0.104
BMI	0.186	0.905	0.010
DIABETES	-0.211	0.421	0.152
DIALYSIS VINTAGE	0.006	0.597	0.073
HLA-MM	0.018	0.654	0.074

Supplemental figure legend:

Supplemental Figure 1. Study flow chart.

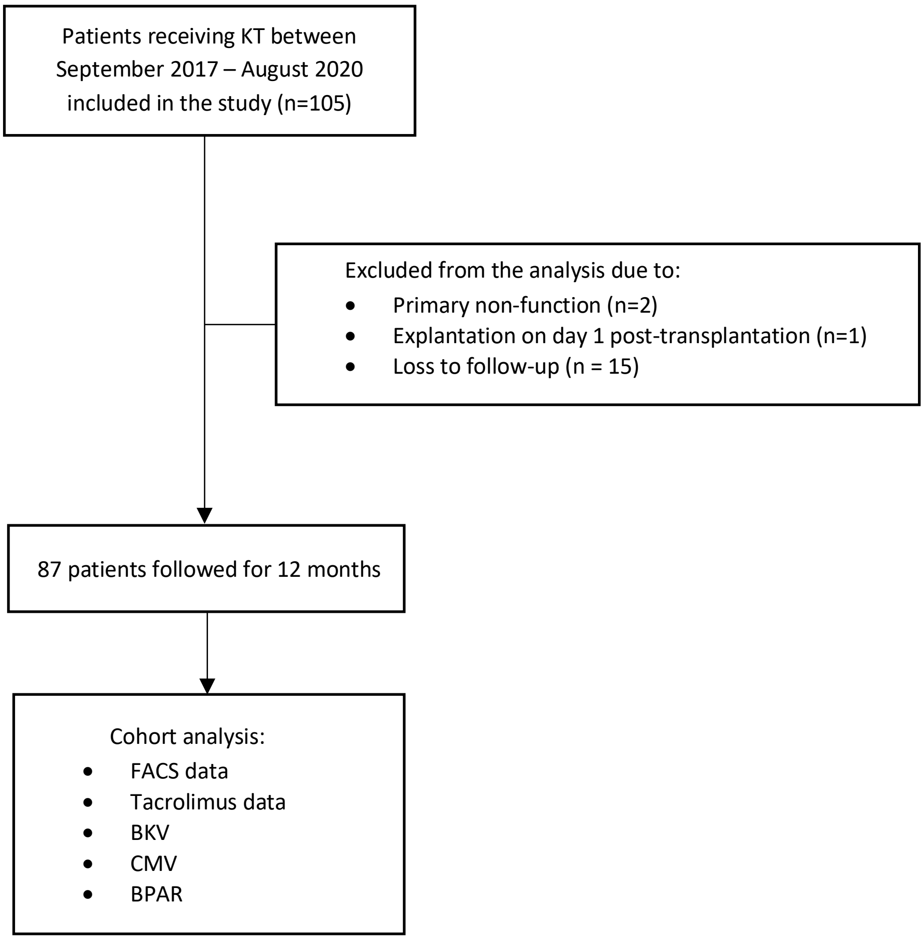

Supplemental Figure 2. Flow cytometry gating strategies.

A: The gating strategy of CD4^{+} regulatory T cells, **B:** CD4^{+} T helper cells shown from a “preKT” sample.

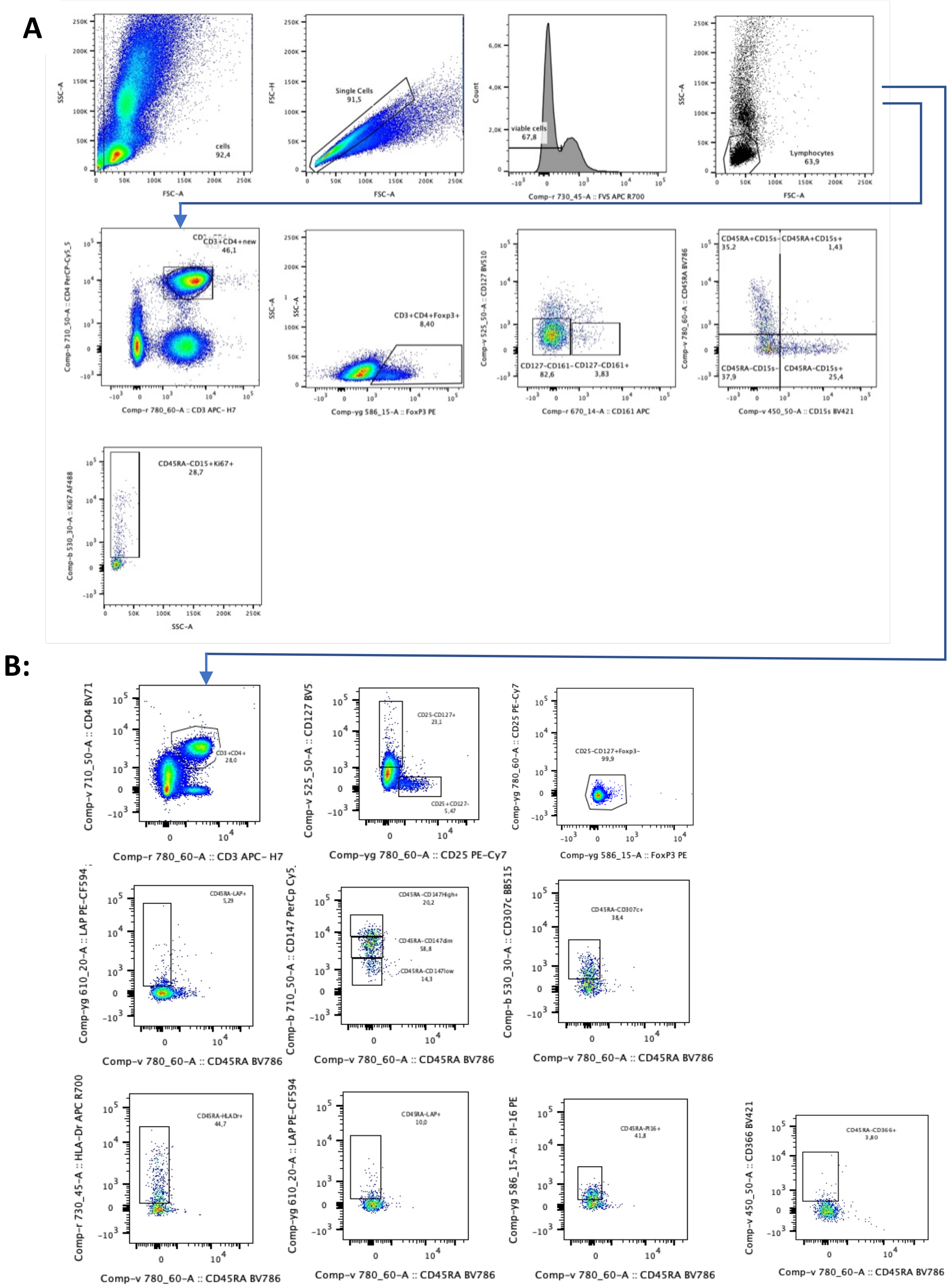

Supplemental Figure 3. Cumulative incidences of outcomes (CMV, BKV, BPAR).
Kaplan-Meier curves were created to visualize the cumulative event-free survival for all outcomes.

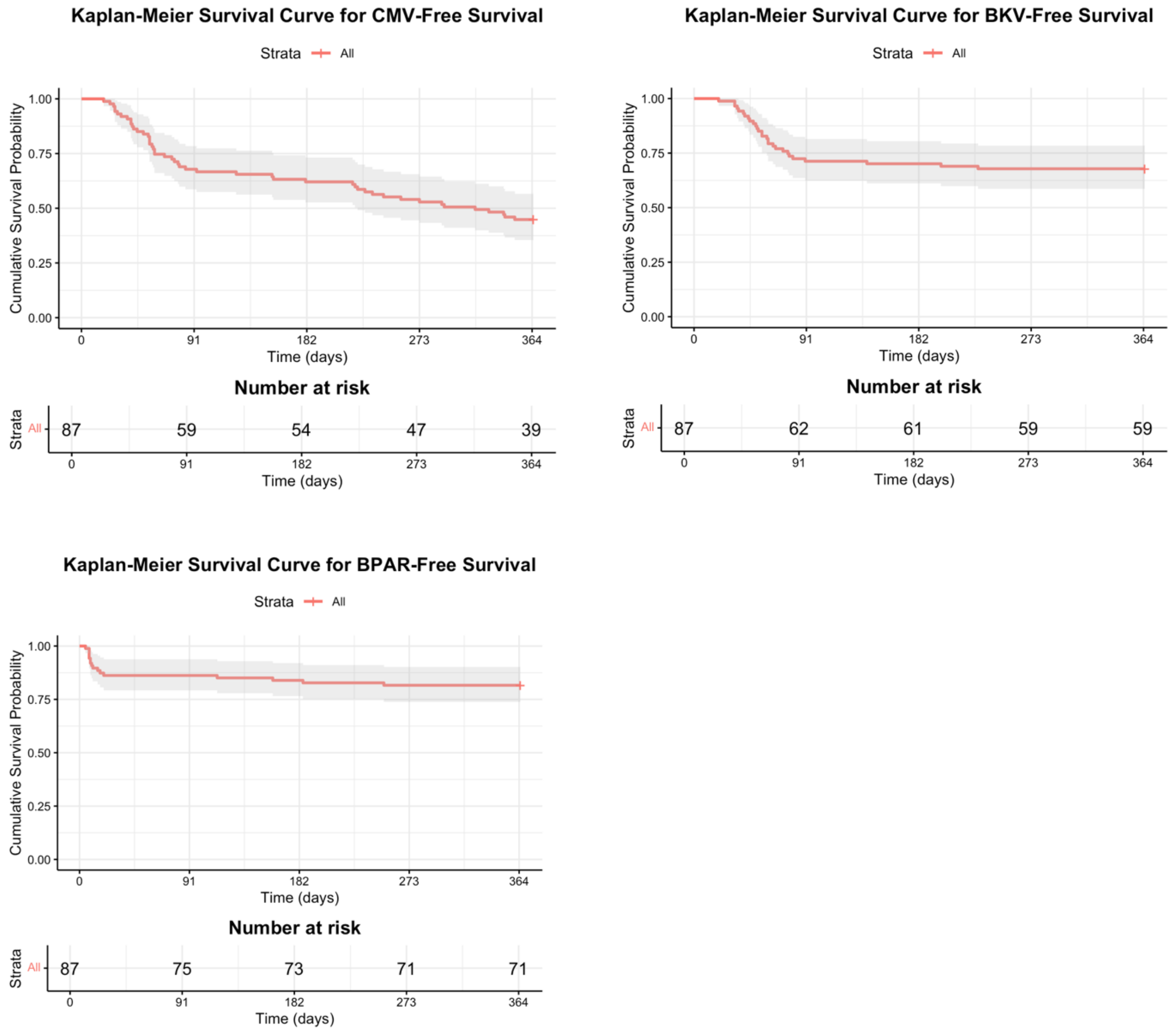

Supplemental Figure 4. The longitudinal change of CD4$^+$ activation markers in effector T cells.
A: Absolute cell counts and **B:** Frequencies of CD4$^+$ T_{eff} subsets are depicted as box blots with median and interquartile ranges between all study visits. Multiple group comparison was done by a mixed-effects analysis; significant results are shown by asterisks (*) p < 0.05, (**) p < 0.01, (****) p < 0.0001.

A

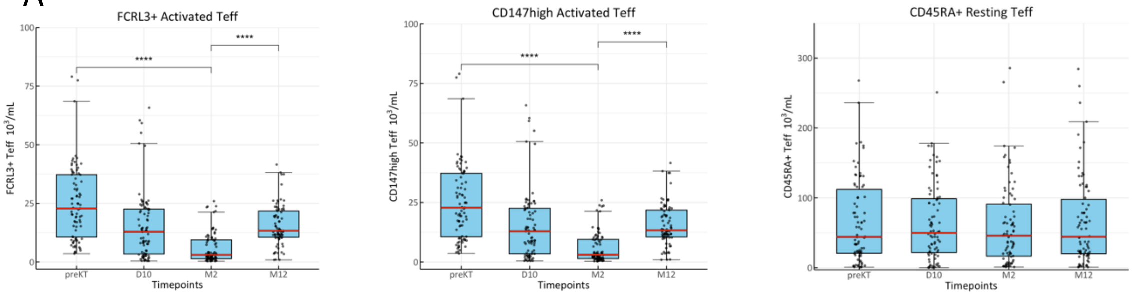

B

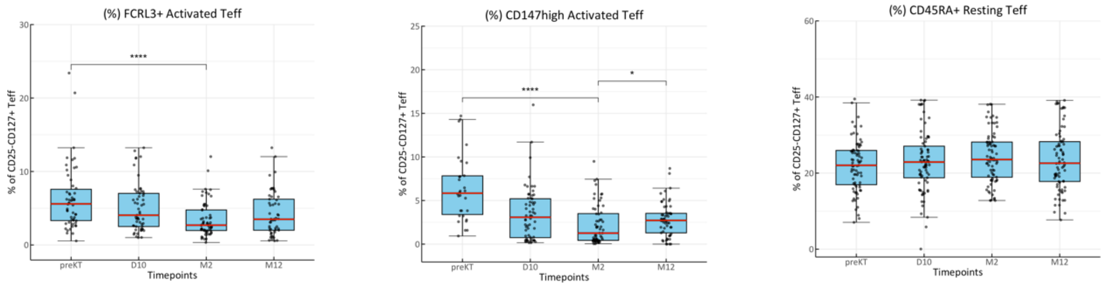

Supplemental Figure 5. The longitudinal change of CD4$^+$ activation markers in regulatory T cells.
A: Absolute cell counts and **B:** Frequencies of CD4$^+$ T_{reg} subsets are depicted as box blots with median and interquartile ranges between all study visits. Multiple group comparison was done by a mixed-effects analysis; significant results are shown by asterisks (**) p < 0.01, (****) p < 0.0001.

A

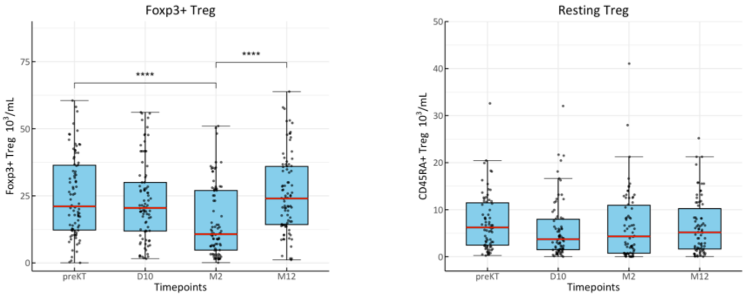

B

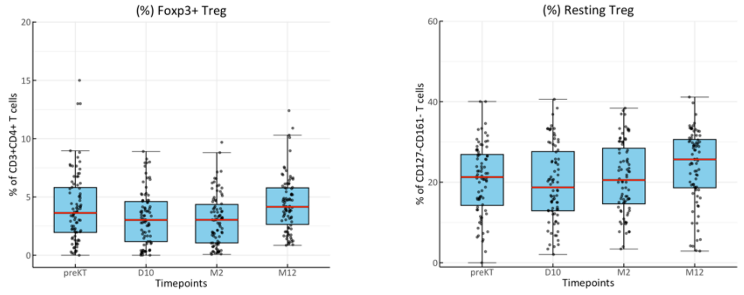

Supplemental Figure 6. Longitudinal changes of CD25⁺Foxp3⁺ T_{reg}, Foxp3⁺ T_{reg} and HLA-DR⁺ T_{eff} by induction agent.

A: Absolute counts and frequencies of CD25⁺Foxp3⁺ T_{reg} demonstrating significant interference of basiliximab with CD25 expression at day 10 and m2. Absolute counts and frequencies of **B:** Foxp3⁺ T_{reg} and **C:** HLA-DR⁺ T_{eff} show a similar longitudinal evolution independent of induction agent.

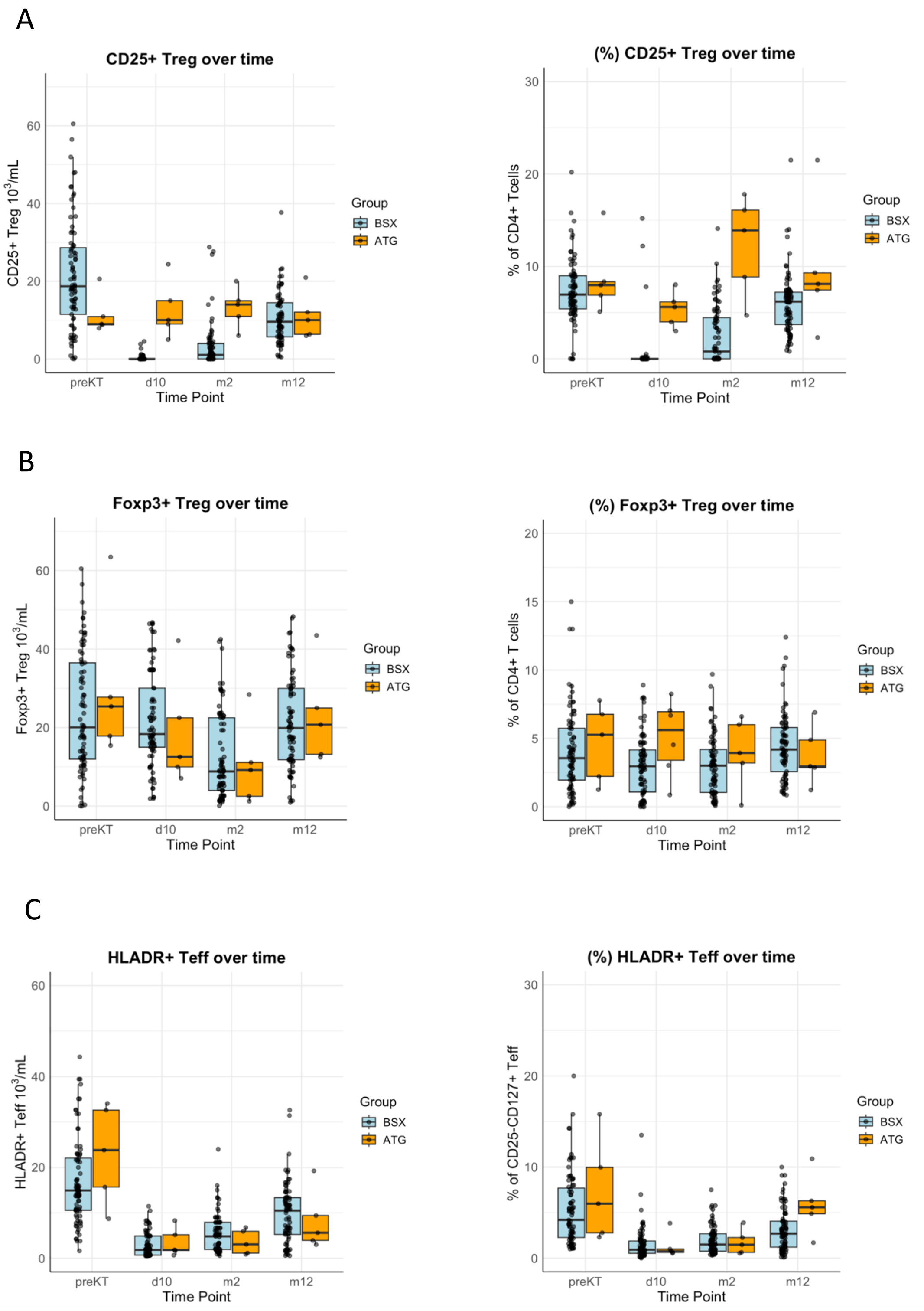

Supplemental Figure 7. Threshold of HLA-DR^{+} T_{eff} count at day 10 to predict BK viremia.
A: Time-dependent ROC curve analysis yielded AUC 0.75, p = 0.002 with determination of a cutoff at 4.71 x 10^3/mL cells (at day 10 to predict future events) by Youden index optimization. **B:** Time-dependent ROC graph derived from the cox regression model.

A

ROC ANALYSIS OF HLA-DR+ TEFF COUNTS				
AUC	p-value	Cutoff (Youden)	Specificity	Sensitivity
0.75	0.001	4.71 (0.48)	0.63	0.85

B

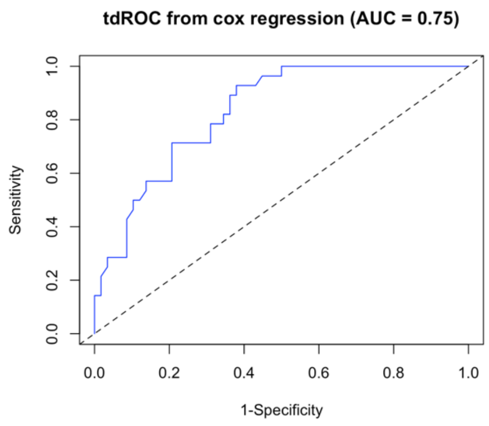

Supplement: Supplementary file 1 [file DataSheet1.pdf]
